# Language task-based fMRI analysis using machine learning and deep learning

**DOI:** 10.3389/fradi.2024.1495181

**Published:** 2024-11-27

**Authors:** Elaine Kuan, Viktor Vegh, John Phamnguyen, Kieran O’Brien, Amanda Hammond, David Reutens

**Affiliations:** ^1^ Centre for Advanced Imaging, The University of Queensland, Brisbane, QLD, Australia; ^2^ ARC Training Centre for Innovation in Biomedical Imaging Technology, The University of Queensland, Brisbane, QLD, Australia; ^3^ Australia Institute for Bioengineering and Nanotechnology, The University of Queensland, Brisbane, QLD, Australia; ^4^ Neurology Department, Royal Brisbane and Women’s Hospital, Brisbane, QLD, Australia; ^5^ Siemens Healthineers, Siemens Healthcare Pty Ltd, Brisbane, QLD, Australia

**Keywords:** task-based fMRI, language, time series, brain activation, machine learning, deep learning

## Abstract

**Introduction:**

Task-based language fMRI is a non-invasive method of identifying brain regions subserving language that is used to plan neurosurgical resections which potentially encroach on eloquent regions. The use of unstructured fMRI paradigms, such as naturalistic fMRI, to map language is of increasing interest. Their analysis necessitates the use of alternative methods such as machine learning (ML) and deep learning (DL) because task regressors may be difficult to define in these paradigms.

**Methods:**

Using task-based language fMRI as a starting point, this study investigates the use of different categories of ML and DL algorithms to identify brain regions subserving language. Data comprising of seven task-based language fMRI paradigms were collected from 26 individuals, and ML and DL models were trained to classify voxel-wise fMRI time series.

**Results:**

The general machine learning and the interval-based methods were the most promising in identifying language areas using fMRI time series classification. The geneal machine learning method achieved a mean whole-brain Area Under the Receiver Operating Characteristic Curve (AUC) of 0.97±0.03, mean Dice coefficient of 0.6±0.34 and mean Euclidean distance of 2.7±2.4 mm between activation peaks across the evaluated regions of interest. The interval-based method achieved a mean whole-brain AUC of 0.96±0.03, mean Dice coefficient of 0.61±0.33 and mean Euclidean distance of 3.3±2.7 mm between activation peaks across the evaluated regions of interest.

**Discussion:**

This study demonstrates the utility of different ML and DL methods in classifying task-based language fMRI time series. A potential application of these methods is the identification of language activation from unstructured paradigms.

## 1 Introduction

Individual variation in functional representation in the cerebral cortex (1, 2) and the potential for re-organisation in the setting of neurological disorders (3, 4) make it crucial to accurately localise eloquent areas of the cortex when surgery that may impinge on these areas is contemplated. Accurate localization plays a key role in guiding the decision to proceed with surgery and in intra-operative surgical guidance aiming to minimize the risk of post-operative neurological deficits. Functional Magnetic Resonance Imaging (fMRI) is a non-invasive method of functional brain mapping that measures the blood oxygen level dependent (BOLD) signal changes in the brain due to changes in regional cerebral blood flow during brain activation (5, 6). It is commonly used for non-invasive pre-surgical mapping across a range of functional domains, including the key domain of language (7–11).

Task-based language fMRI is a conventional approach for presurgical language mapping. It achieves language lateralization and localization that is concordant with gold standard methods, such as direct cortical stimulation and the Wada test (12–14). In task-based language fMRI, the subject completes a specific language task arranged in a paradigm comprising of blocks with task performance interleaved with blocks during which a control or baseline task is performed, (11, 15). Such designs facilitate statistical analysis using the General Linear Model to identify brain areas activated during the performance of the language task (16, 17). In these areas, the time course of the fMRI signal resembles the temporal profile predicted from the structure of the task (i.e., the task regressor). Clinical application of task-based fMRI requires the patient to understand and perform the task paradigm, potentially hampering its use in patient groups such as young children or those with deficits in comprehension, memory or attention that interfere with task performance.

This limitation of task-based paradigms can be overcome by using unstructured, continuous paradigms such as naturalistic fMRI (18–22). Naturalistic paradigms are less demanding in terms of patient compliance and mimic everyday activities insofar as they may only involve passive viewing of a movie or video (18, 20). However, unlike task-based fMRI, naturalistic fMRI has no obvious task regressor of interest. In previous studies, regressors have been defined by manually labelling the movie stimulus to identify features that are considered a priori to engage specific cognitive processes (23, 24). The labelling procedure is subjective and likely to vary with the expertise of the reviewer. A more direct alternative approach would be to extract the regressor(s) of interest from the temporal profiles of already-defined functional systems. One way to investigate the feasibility of this approach is to validate extracted temporal profiles using task-based paradigms in which the temporal profiles are known.

Machine Learning (ML) and Deep Learning (DL) methods have already been applied to fMRI analysis [See (25) for a general review of the applications of ML and DL in fMRI data analysis]. ML and DL methods are data-driven and their potential use for the classification of fMRI time series in keeping with their ability to classify time series in other application domains (26–28). However, the application of ML/DL methods to voxel-wise time series fMRI data (i.e., 1D data) have not been considered to date. The best ML/DL approach for learning task regressors are also yet to be determined.

To answer these questions, we first investigated the ability of different ML and DL algorithms to detect language activation by task-based language fMRI paradigms in individuals. We evaluated different types of ML and DL algorithms using a range of clinically relevant performance metrics including Area Under the Receiver Operating Characteristic curve (AUC), the Dice coefficient and the Euclidean distances between corresponding activation peaks identified by the ML or DL methods and those identified by the gold standard, the General Linear Model. This approach enabled us to determine the ML/DL methods that identify areas of language activation corresponding to the gold standard. This result serves as the foundation for future work to extract the task regressors from naturalistic paradigms using ML/DL methods.

## 2 Materials and methods

### 2.1 Participants

The study was approved by the Royal Brisbane and Women’s Hospital Human Research Ethics Committee. All participants provided written informed consent. The study comprised of 26 individuals (20 healthy participants and 6 epilepsy patients; mean age 40, range 21–71 years, 13 females). Head motion for each individual across each language language paradigm was accessed using Framewise Displacement (FD) (29). Framewise Displacement for the 26 participants across all language paradigms were found to be within the acceptable range (Mean FD =0.12±0.06 mm, maximum FD less than 0.4 mm). All participants’ primary language was English and handedness was assessed using the Edinburgh Handedness Inventory (EHI) Questionnaire.

### 2.2 Task design

All participants underwent seven task-based language fMRI scans in a single session. The paradigms utilized a block design with task blocks interleaved with control blocks. Participants were provided with training for each task before the scanning session and at the beginning of each task, instructions were presented on screen followed by 10 s of dummy scans, during which a black screen was presented. Dummy scans were excluded from the analysis. At the end of each task, a black screen was also presented for 10 s to allow for signal stabilisation. See Table 1 for more information.

**Table 1:** Task paradigms.

Paradigm	Duration	No. blocks	Details of each block	Participant instructions
Sentence Completion (SC)	4:20	6 task, 6 control	4 incomplete sentences or garbled sentences, presented for 5 s each	Participants were instructed to think of the word that completes the sentence during task blocks
Silent Word Generation (SWG)	4:20	6 task, 6 control	2 alphabets or symbols, presented for 10 s each.	Participants were instructed to silently think of as many words as possible beginning with the alphabet shown on the screen during task blocks
Rhyming (R)	4:20	6 task, 6 control	5 sets of two words or symbols, presented for 4 s each.	Participants were instruct to press a button when the words or symbols presented to them rhyme or match.
Object Naming (ON)	4:20	6 task, 6 control	6 images or symbols, presented for 3.34 s each	Participants were instructed to think of the name of the object presented on screen during the task blocks.
Antonym Generation (AG)	3:00	4 task, 4 control	10 words or fixation crosses presented for 2 s each.	Participants were told to think of the antonym of the word during the task blocks.
Passive Story Listening (PSL)	4:20	6 task, 6 control	One segment of the story or garbled audio, played for 20 s	Participants were instructed to close their eyes and pay attention to the audio story during the task blocks
Sentence Completion Listening (SCL)	4:20	6 task, 6 control	4 audio sentences (with the beep indicating the end of the sentence) or garbled sentences presented for 5 s each.	Participants were asked to think of the word that completes the sentence at the end of the beep during the task blocks.

For each task, participants were instructed to respond covertly during the task blocks (i.e., to think of the responses and not to speak out loud) and to fixate on a fixation point presented on the screen during control blocks.

Task stimuli were presented with E-prime 3.0 software (Psychology Software Tools Inc., version 3.0) and participants were given MRI-safe active noise-cancelling headphones. Participants who required vision correction were encouraged to wear contact lenses or MRI-safe vision correction lenses were used.

### 2.3 Image acquisition

Data were collected using a Siemens Magnetom 3T Prisma scanner (Siemens Healthcare, Erlangen, Germany). BOLD Functional images were acquired using an Echo Planar (EPI) sequence and standard 64 channel head coil. (TR = 2,000 ms, TE = 23 ms, Flip angle = 90 Degrees, FoV = 210 mm, Resolution =70×70, 42 Axial Slices, Voxel size =3×3×3 mm). Whole brain T1 MPRAGE structural images were also acquired (TR = 1,900 ms, TE = 256 mm, Flip Angle = 9 Degrees, FoV = 256 mm, Resolution =256×256, 192 Axial Slices, Voxel size =1×1×1 mm).

### 2.4 Pre-processing of fMRI data

Data were analysed using Statistical Parametric Mapping SPM12 software (30, 31). The steps included slice timing correction, realignment (realigning images to the mean image functional image across all tasks for each participant), co-registration to structural T1 image, spatial normalization to Montreal Neurological Institute (MNI) space (re-sampled to 3×3×3 mm) and smoothing using a full-width half maximum (FWHM) Gaussian kernel of 6 mm.

### 2.5 Task-based language fMRI activation

For each participant, first-level task-based language activation maps were derived for each of the seven tasks. Pre-processed voxel-wise fMRI time series data were modeled with the General Linear Model using SPM12 software. General linear modelling was performed with defined task regressors and covariates. The former were obtained by convolving the box-car stimulus function (contrasting task and control conditions) of each task with the canonical Haemodynamic Response Function (HRF). Covariates or nuisance regressors were the six motion parameters from the motion correction step during pre-processing. High pass filtering with a cut off of 128s and a first-level autoregressive model, i.e., AR(1) were employed.

**Figure 1:**
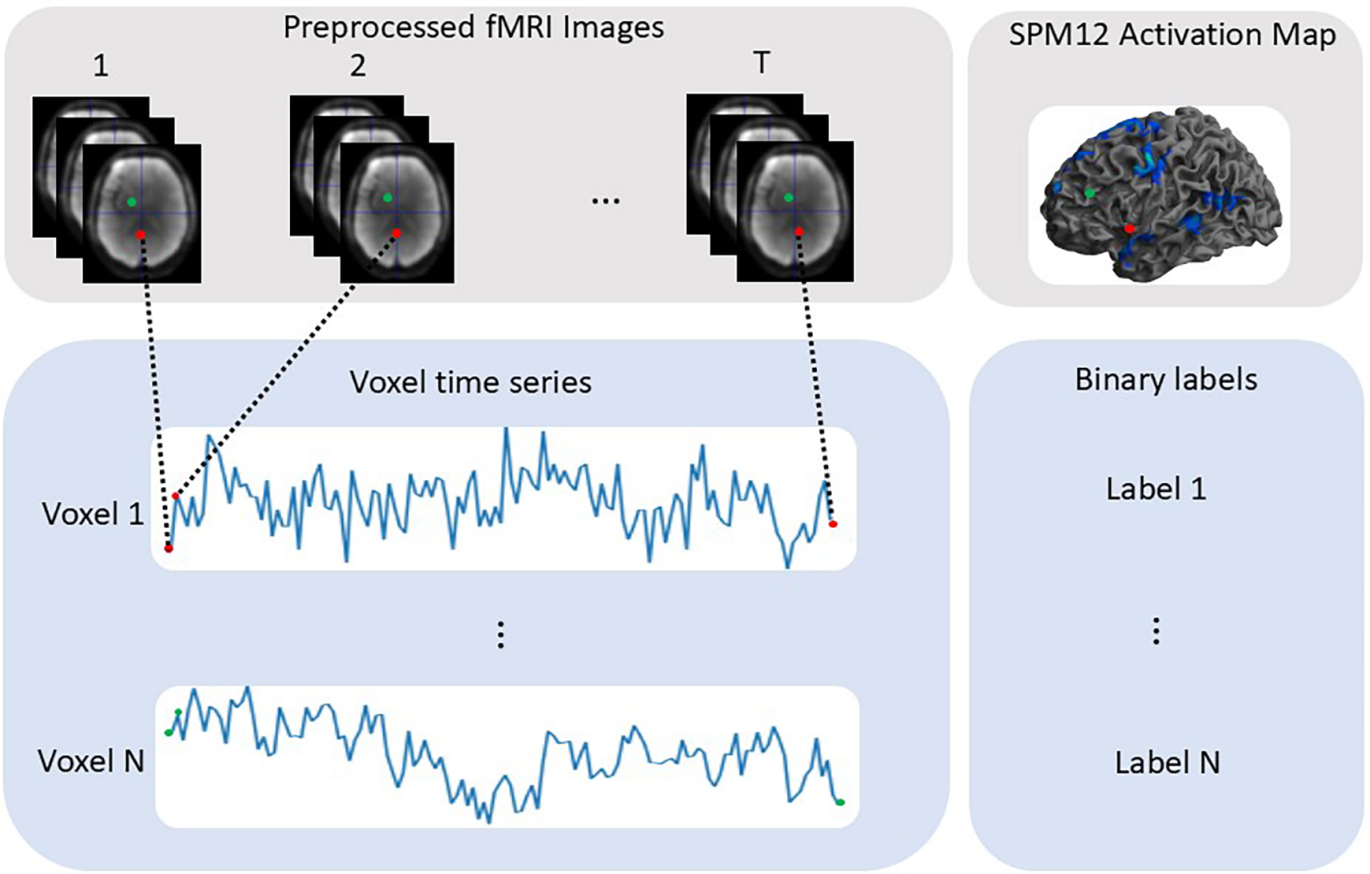
Illustrated are the steps to obtain task-based language activation maps. Task paradigm stimulus function was convolved with the hemodynamic response function to get task regressors. Raw fMRI images were preprocessed, and together with task and nuisance regressors activation maps are produced using Genearl Linear modelling.

A threshold of p<0.001, uncorrected for multiple comparisons, was applied to the t-statistic images to generate language activation maps which were binarised (activated vs non-activated) for machine learning analysis. The process of deriving task-based language activation maps is illustrated in Figure 1.

### 2.6 Training and test pipeline

Data analysis with machine learning and deep learning comprises of training and testing stages. A set of carefully curated training data is first provided to ML or DL algorithms, allowing patterns to be extracted from the data, resulting in a trained model. Test set(s) are then used to evaluate the performance of the ML/DL model.

#### 2.6.1 Training set

**Figure 2:**
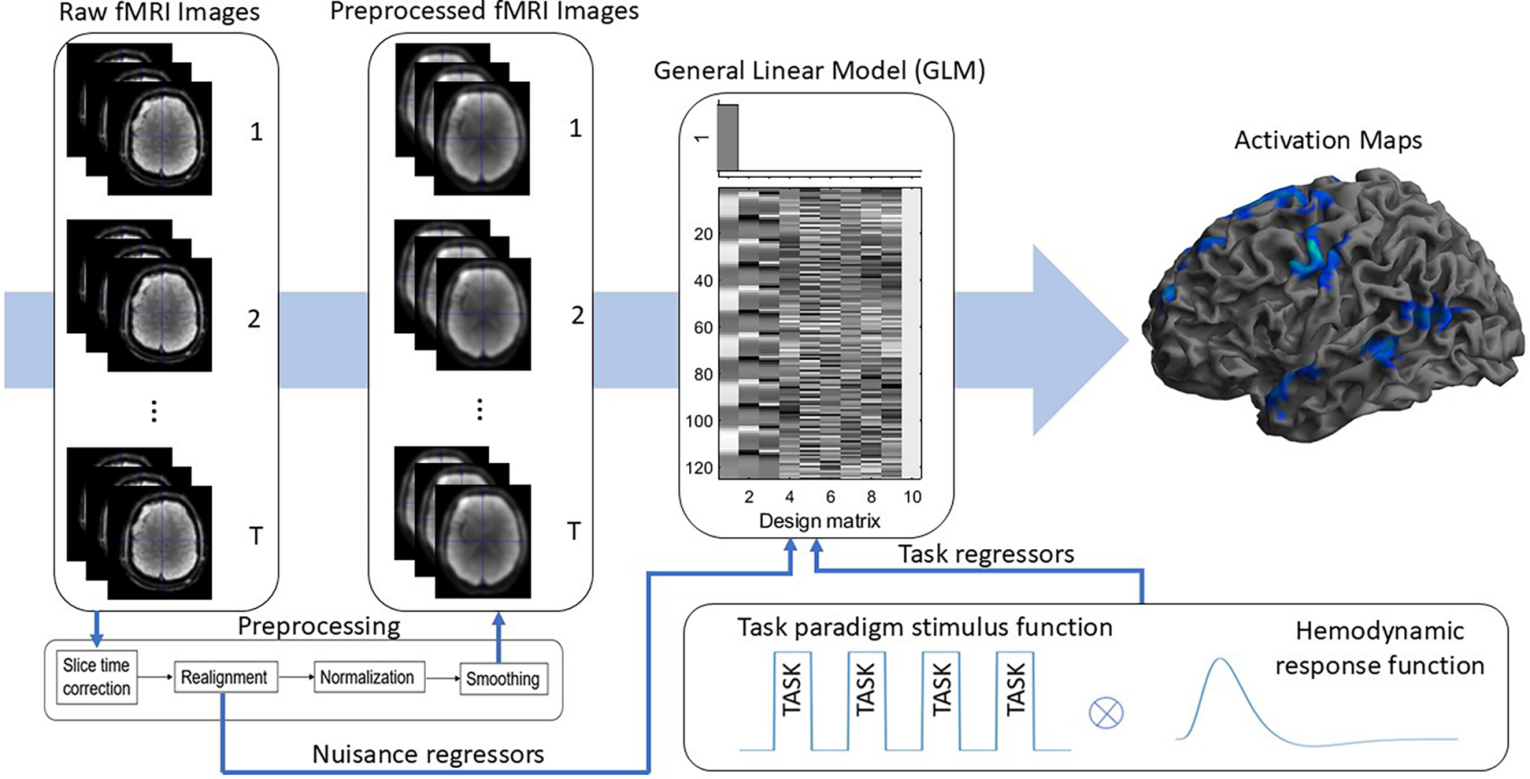
Illustrated are the steps to extract voxel time series data and corresponding binary labels. Task-based language activation maps were used to define the 0 and 1 labels.

To construct the training set, pre-processed fMRI voxel time series of 14 healthy participants across 6 tasks (SC, SWG, R, ON, PSL, SCL) were extracted and labelled as activated (label 1) or non-activated (label 0) to yield binarised activation maps for each paradigm. The classification problem to be solved by the ML/DL methods was therefore to classify whether a fMRI voxel time series was activated or non-activated. The process of extracting voxel-wise fMRI time series and labelling is illustrated in Figure 2. Data from the AG tasks were excluded from the training set because the length of the voxel time series differed from that of the other tasks; however, the AG task was included in the test set, see Section 2.6.2).

Reflecting individual variation in brain function, the number of activated and non-activated fMRI voxel time series varied across participants and tasks. To balance the training set so that the numbers of activated and non-activated time series was equal, and to equalize the contribution of samples from each participant and each task, the participant and task paradigm with the smallest number of activated voxels was identified, which corresponded to 29 fMRI time series samples. The number of activated fMRI voxel time series and non-activated voxel time series for the remaining participants and tasks was randomly sampled, without replacement, to match the smallest number of activated voxels (i.e., resulting in 58 samples from each task paradigm for each participant, with the number of activated voxel time series and non-activated voxel time series from each participant totaling up to 348).

The resulting training set had 4,872 samples, of which 2,436 samples were labelled 1 and 2,436 samples were labelled 0.

#### 2.6.2 Test set

For the test set, time series from all brain voxels in 6 healthy and 6 epilepsy patients for 6 tasks (SC, SWG, R, AG, ON, SCL) were extracted and labelled according to the binarised language activation maps. A combination of healthy participants and epilepsy patients were included in the test set to ensure diversity in the test data. The PSL task was excluded from analysis because there was little activation in most participants. To determine whether the trained models could be used to analyse unseen data with fMRI time series of different lengths, the time series for the AG task was padded with the final time point in the fMRI time series to match the length of fMRI time series of other tasks.

An average of 66,036 samples was extracted from each task in each participant. The voxel locations of each sample were saved and later used to reconstruct ML/DL activation maps. See Table A1 in Appendix (Section A) for the number of samples that were extracted for each participant and each task.

#### 2.6.3 Choice of machine learning and deep learning algorithms

Several types of machine learning and deep learning algorithms have been proposed for time series classification. They can be categorised into general machine learning, dictionary-based, distance-based, feature-based, frequency-based, interval-based, kernel-based, shapelet-based, hybrid or ensemble-based and deep learning methods. The different ML/DL categories and methods can be found in the SKTIME library (version 13.4) (32, 33). A separate category was defined, namely the general machine learning (GML) category which includes ML methods that were conventionally developed to solve non-time series problems. These methods can be found in the Scikit-Learn library and the Rotation Forest algoritm falls into this category (34, 35).

A representative algorithm from each category is chosen based on the literature and evaluated to determine whether a particular category of classification algorithms was more suited to task-based language fMRI analysis. Table 2 shows the algorithms from each category. Default hyper-parameters were used to evaluate each of the chosen algorithms. While hyper-parameter tuning is largely discussed in the machine learning community, Probst et al. (36) evaluated the impact of hyper-parameter tuning on six different ML algorithms and showed that AUC at most improves by 10% by optimizing of hyper-parameters. Default hyper-parameters are also useful starting points to ML algorithm evaluation and generally work well across different problems (35). The use of default parameters also ensures reproducibility of results.

**Table 2:** Algorithms evaluated from different categories.

Category	Algorithm
General Machine Learning (GML)	Rotation Forest (RotF) (35)
Dictionary-based	Word Extraction for Time Series Classification (WEASEL) (37)
Feature-based	Time Series Feature Extraction based on Scalable Hypothesis Tests (TSFresh) (38)
Frequency-based	Random Interval Spectral Ensemble (RISE) (39)
Interval-based	Supervised Time Series Forest (sTSF) (40)
Kernel-based	Ensemble of RandOm Convolutional KErnel Transform transformers (ARSENAL) (41)
Deep-Learning (DL)	Inception Time (Inception) (42)

To choose the algorithms to be tested, we first identified the most recent algorithms to be developed in each category. The literature was then reviewed to determine their performance in comparison with algorithms previously used to benchmark time series classification performance in terms of accuracy and computational efficiency (28). The algorithms chosen for comparison from each category were trained on high performance computing clusters with different memory requirements depending on the algorithm. Algorithms that required more than 24 h or 128 GB of memory on a single core for training were excluded from selection. Because long test times can be overcome by testing multiple batches of data in parallel, we did not consider this in algorithm selection. See Appendix (Section A) for further details on the justification of machine learning algorithm choices.

This study focused on time series classification methods that build on traditional machine learning methods because of their proven success in other application domains involving time series datasets (See (28) for more). While deep learning methods have great success in many domains, DL-based methods often require more data to train and run the risk of over-fitting. Deep learning networks are also more complex, which often makes interpretation challenging.

#### 2.6.4 Performance evaluation

Measures used to assess algorithm performance were Area Under the Receiver Operating Characteristic Curve (AUC), Dice coefficient and Euclidean distance(s) between activation peaks identified with SPM and ML/DL. These were calculated for each participant according to language paradigm and ML/DL algorithm.

AUC was calculated for the whole brain and both the Dice coefficient and Euclidean distance were calculated using 12 language-related regions from both left and right hemispheres defined using parcellations from (43). Of the 12 language-related regions of interest, those for which at least one test participant showed >50 percent overlap between the region and the area of task-based activation were selected, yielding 25 regions across 6 language paradigms.

AUC values were calculated from Receiver Operating Characteristic (ROC) curves which plot the True Positive Rate vs. False Positive Rate for each test participant and task at different probability thresholds. The Dice coefficient was calculated using: $Dice=\frac{2|A\cap B|}{\left|A|+|B\right|}$ , where A corresponds to activated voxels identified by SPM and B corresponds to ML/DL activated areas. AUC and Dice coefficient values range from 0 to 1, with 1 indicating that a ML/DL algorithm performs perfectly in classifying test samples (i.e., full overlap between ML/DL and task-based activation maps. We categorised a Dice coefficient of 0.00–0.19 as low overlap, 0.20–0.39 as low-moderate overlap, 0.40–0.59 as moderate overlap, 0.60–0.79 as moderate-high overlap and 0.80–1.00 as high overlap, in keeping with categories defined by (44).

The Euclidean distance between peaks within language-related regions was also evaluated as peak location is of potential clinical importance in neurosurgical decision-making. In each selected language-related region, the distance between the highest SPM activation peak and every ML/DL activation peak was calculated and the shortest distance reported in millimeters (mm). We also assessed whether the peaks identified by ML/DL and by SPM were in the same or different gyri by co-registering the activation maps to each participant’s T1 MPRAGE structural image.

A Kruskal-Wallis test with post hoc pairwise comparisons using the Dunn test (p-value corrected for multiple comparisons using the Holm–Bonferroni method) were performed to identify if there were significant differences between the mean AUC, Dice coefficient and Euclidean distance between peaks for different ML/DL methods. The threshold for statistical significance was set at p<0.05.

## 3 Results

### 3.1 Activation maps

**Figure 3:**
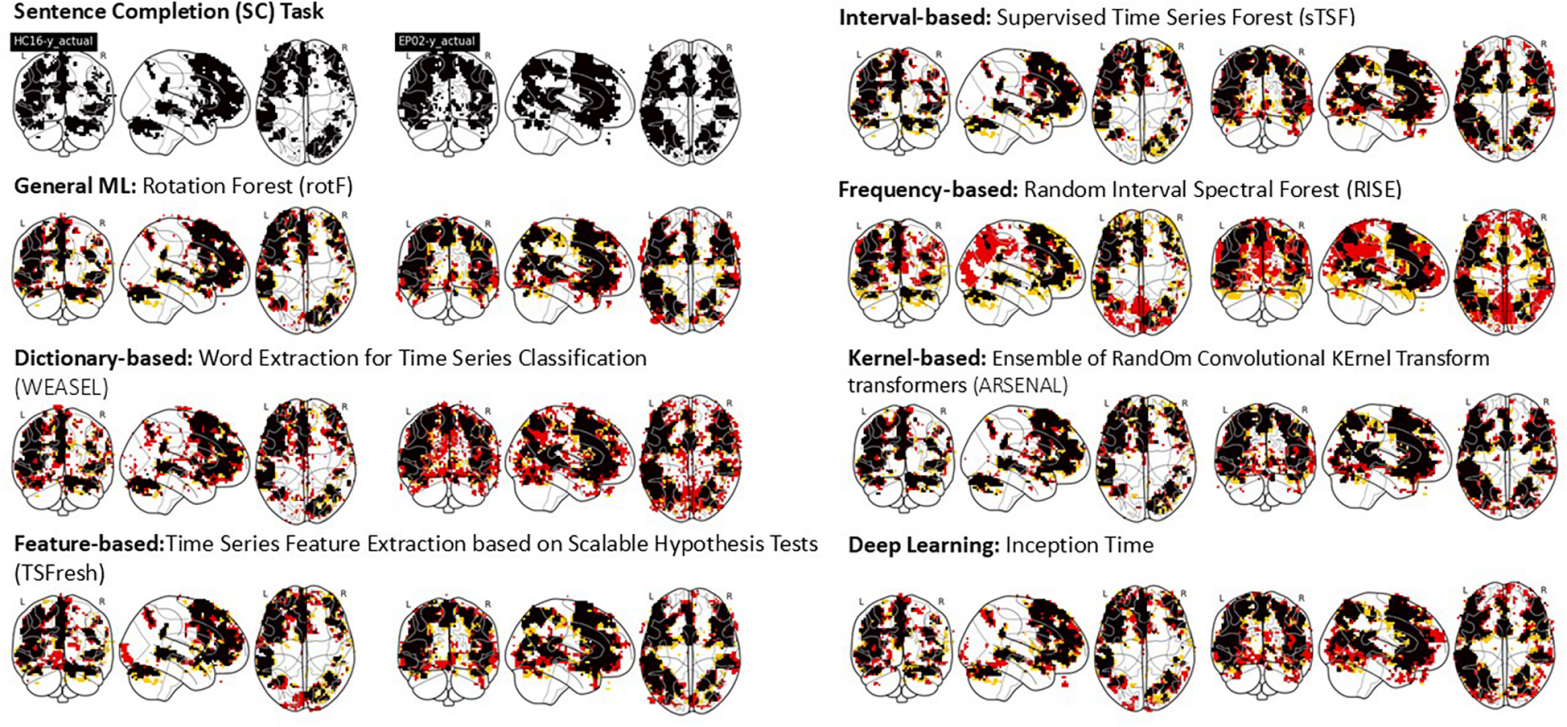
This figure shows the overlap between SPM activation maps vs. evaluated ML/DL activation maps of two test participants (A healthy participant, LHS under each algorithm title and an epilepsy patient, RHS under each algorithm title) for a single single language task - Sentence Completion (SC). Black - Overlap, yellow - SPM activation, Red - ML/DL activation.

To illustrate how well each method performs, Figure 3 shows the overlap between activation areas found by SPM and each ML/DL algorithm studied for the Sentence Completion (SC) task in two test participants (p<0.001 uncorrected). The black areas denote the overlap between SPM and ML/DL activation, yellow areas denote activated areas found only by SPM and red areas denote activated areas only found by each ML/DL methods. Activated areas identified by SPM are shown in the upper left row of the figure. Reflecting the expressive and receptive language components of the SC task, activation can be seen in frontal and temporal lobes in both participants; bilateral activation was observed.

Activated voxels identified by the ML/DL algorithms occurred in clusters and a qualitatively good level of overlap (as indicated by the ratio of black areas compared to red and yellow areas) was observed for most methods except for the frequency-based and dictionary-based methods. The frequency-based method shows areas that are not found to be activated by SPM (red areas in the posterior brain, including occipital and parietal areas). A number of scattered small activated areas were identified by the dictionary-based method, although the main activation clusters were still identified.

### 3.2 Whole-brain AUC

**Figure 4:**
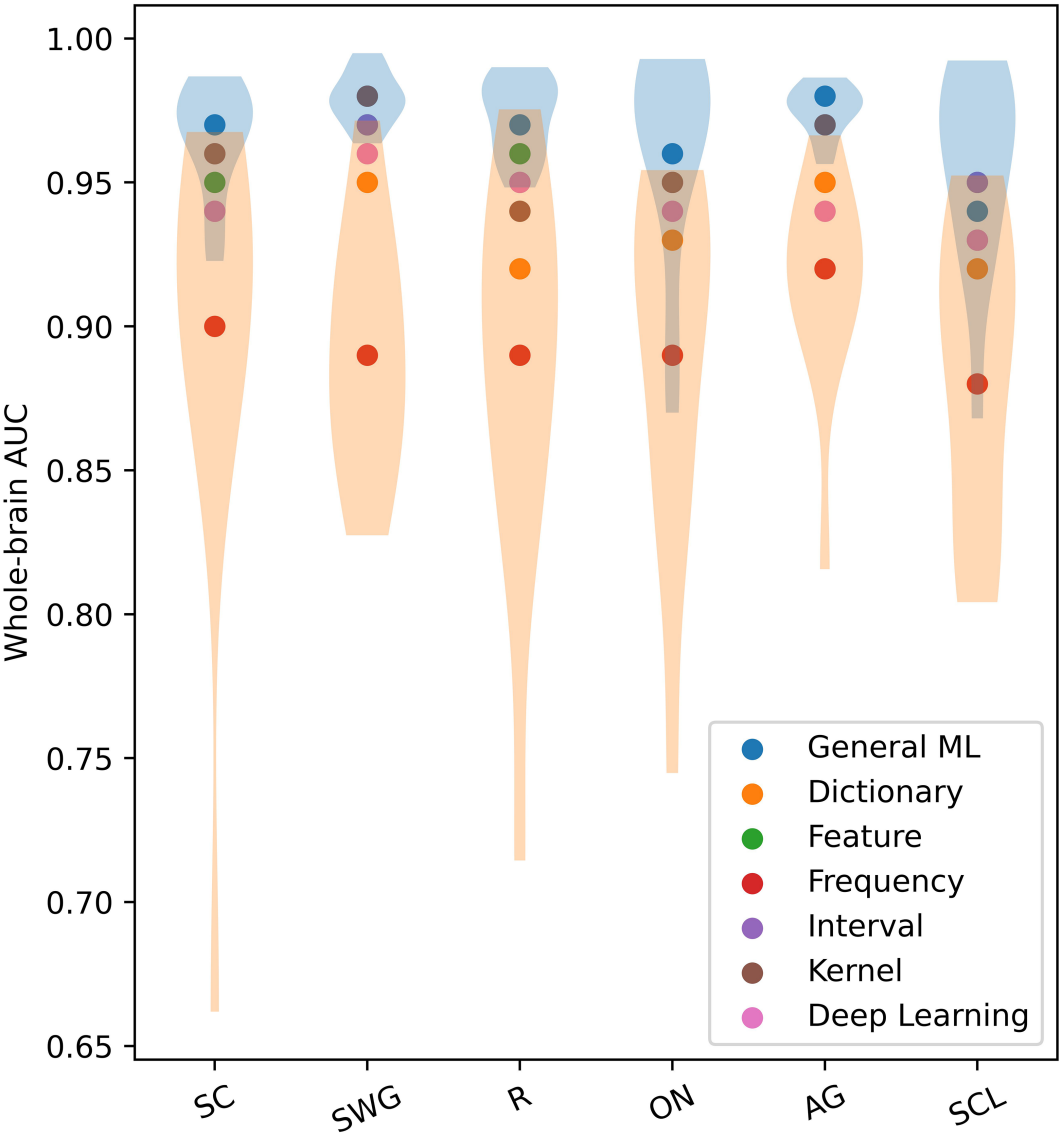
Mean whole-brain AUC values across test participants of different ML/DL categories, by language paradigm (as a scatter plot with violin plots showing the distribution of AUC values for the best and worse performing ML/DL methods for each language paradigm. Blue - best, Orange - worst).

The scatter plot in Figure 4 shows the mean whole-brain AUC values across test participants of different ML/DL categories and language paradigms. The violin plots show the distribution of AUC values across test participants for the best and worse performing ML/DL categories of each language paradigm. Blue violin plots show the distribution of AUC values for the best performing ML/DL method and orange violin plots show the distribution of AUC values for the worst performing ML/DL method. Table 3 shows the values associated with the scatter plot in Figure 4.

**Table 3:** Table of mean whole-brain AUC values across test participants by language paradigm, associated with scatter plot in Figure 4.

	SC	SWG	R	ON	AG	SCL	Mean ± SD
General ML	0.97	0.98	0.97	0.96	0.98	0.94	0.97±0.03
Dictionary	0.94	0.95	0.92	0.93	0.95	0.92	0.94±0.04
Feature	0.95	0.97	0.96	0.95	0.97	0.95	0.96±0.04
Frequency	0.9	0.89	0.89	0.89	0.92	0.88	0.89±0.06
Interval	0.96	0.97	0.95	0.95	0.97	0.95	0.96±0.03
Kernel	0.96	0.98	0.94	0.95	0.97	0.93	0.95±0.04
Deep Learning	0.94	0.96	0.95	0.94	0.94	0.93	0.94±0.04

Mean whole-brain AUC values for different ML/DL categories and language paradigms exceeded 0.8 with the GML method achieving the highest AUC values for 5 of 6 of the language paradigms (highlighted in blue in Table 3). For the SWG paradigm, both the GML and kernel-based methods achieved the highest AUC value of 0.98. AUC values for the GML method ranged between 0.94 to 0.98, with values being highest for SWG and AG (0.98). The highest mean AUC value across language paradigms was achieved by the GML method (0.97±0.03; Table 3). The frequency-based method consistently ranked the lowest in whole-brain AUC values, with values ranging from 0.93 to 0.96 (Mean AUC: 0.89±0.06; Table 3).

The mean AUC differed significantly between ML categories (Kruskal-Wallis Test, H(6)=131.1, $p=7.34\times 10^{-26}$). On post hoc testing, the mean AUC of the GML method was found to be significantly higher than that of the dictionary, frequency and DL-based methods. The mean AUC of the frequency-based method was found to be significantly lower than that of all other evaluated methods.

When evaluated across the healthy participant and epilepsy patient groups, the GML method consistently ranks the top three when evaluated across the different language paradigms. This occurs in 89% of cases for healthy participant group and 72% for the epilepsy patient group. The GML method ranks the top two in 75% of cases for healthy participant group and 64% for the epilepsy patient group, and ranks the highest for 50% of cases for healthy participant group and 42% for the epilepsy patient group. In contrast, the frequency-based method ranks lowest among both groups, with 83% of cases for healthy participants and 86% for epilepsy patients across the evaluated language paradigms. This suggests consistent performance of ML/DL methods between healthy participants and epilepsy patients.

### 3.3 Dice coefficients of language regions

**Figure 5:**
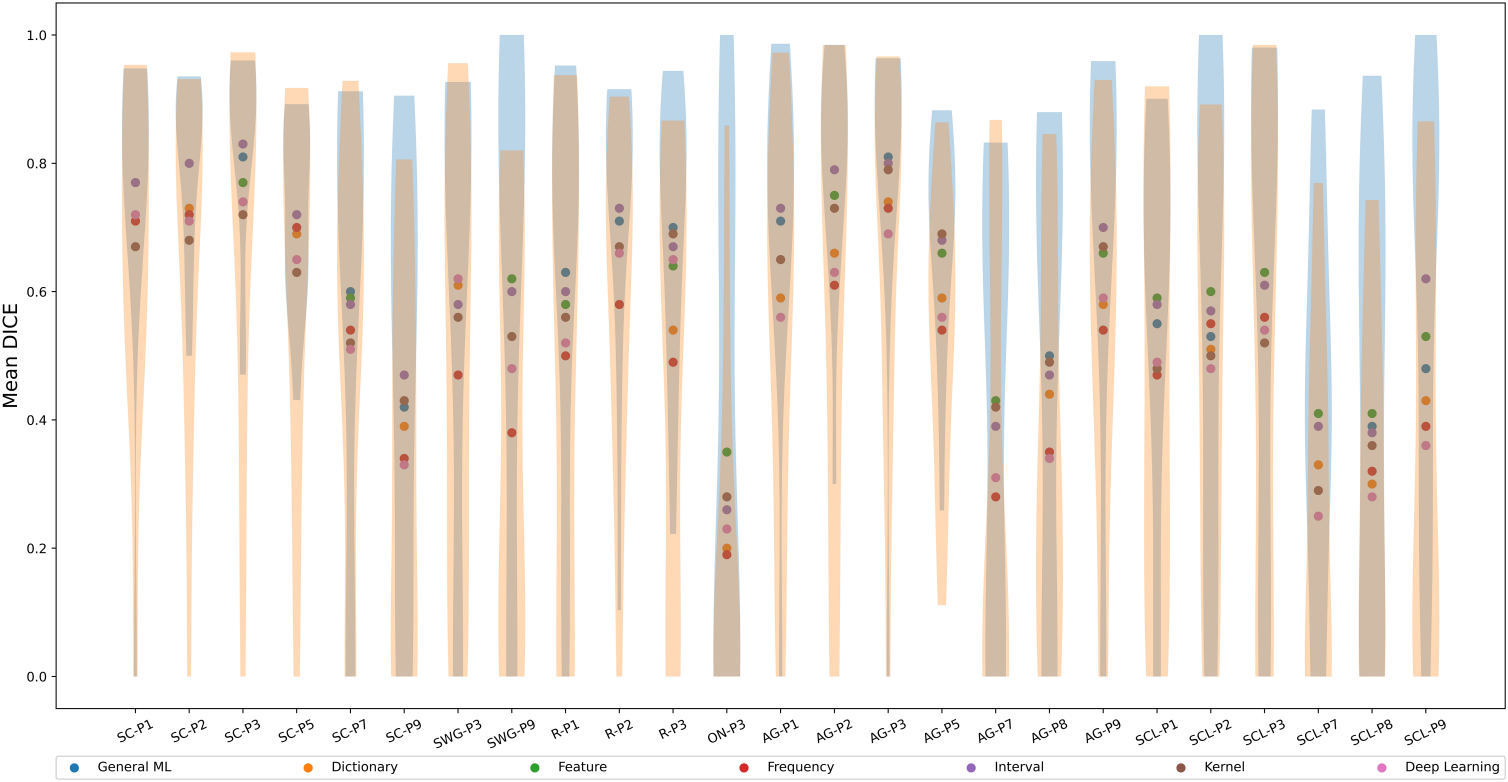
Mean Dice coefficient across test participants of different ML/DL categories, by language regions of interest (as a scatter plot with violin plots denoting the distribution of Dice coefficient values for the best and worse performing ML/DL methods for each language paradigm. Blue - best, Orange - worst).

Figure 5 shows the mean Dice coefficients (across test participants) for each ML/DL category in different language-related regions. The violin plots show the distribution of Dice coefficients across test participants for the best and worse performing ML/DL categories of each language region of interest. Blue violin plots show the distribution of Dice coefficients for the best performing ML/DL method and orange violin plots show the distribution of Dice coefficient values for the worst performing ML/DL method. Table 4 shows the values associated with Figure 5.

**Table 4:** Table of mean Dice coefficient across test participants by language language regions of interest, associated with Figure 5.

	SC-P1	SC-P2	SC-P3	SC-P5	SC-P7	SC-P9	SWG-P3	SWG-P9	R-P1	R-P2	R-P3	ON-P3	AG-P1	AG-P2	AG-P3	AG-P5	AG-P7	AG-P8	AG-P9	SCL-P1	SCL-P2	SCL-P3	SCL-P7	SCL-P8	SCL-P9	Mean ± SD
General ML	0.77	0.8	0.81	0.72	0.6	0.42	0.62	0.6	0.63	0.71	0.7	0.28	0.71	0.75	0.81	0.68	0.42	0.5	0.67	0.55	0.53	0.61	0.33	0.39	0.48	0.6±0.34
Dictionary	0.71	0.73	0.77	0.69	0.51	0.39	0.61	0.48	0.56	0.66	0.54	0.2	0.59	0.66	0.74	0.59	0.39	0.44	0.58	0.49	0.51	0.61	0.33	0.3	0.43	0.54±0.33
Feature	0.71	0.72	0.77	0.72	0.59	0.43	0.62	0.62	0.58	0.73	0.64	0.35	0.65	0.75	0.79	0.66	0.43	0.49	0.66	0.59	0.6	0.63	0.41	0.41	0.53	0.6±0.34
Frequency	0.71	0.72	0.72	0.7	0.54	0.34	0.47	0.38	0.5	0.58	0.49	0.19	0.65	0.61	0.73	0.54	0.28	0.35	0.54	0.47	0.55	0.56	0.25	0.32	0.39	0.5±0.35
Interval	0.77	0.8	0.83	0.72	0.58	0.47	0.58	0.6	0.6	0.73	0.67	0.26	0.73	0.79	0.8	0.68	0.39	0.47	0.7	0.58	0.57	0.61	0.39	0.38	0.62	0.61±0.33
Kernel	0.67	0.68	0.72	0.63	0.52	0.43	0.56	0.53	0.56	0.67	0.69	0.28	0.65	0.73	0.79	0.69	0.42	0.49	0.67	0.48	0.5	0.52	0.29	0.36	0.36	0.56±0.35
Deep Learning	0.72	0.71	0.74	0.65	0.51	0.33	0.62	0.48	0.52	0.66	0.65	0.23	0.56	0.63	0.69	0.56	0.31	0.34	0.59	0.49	0.48	0.54	0.25	0.28	0.36	0.52±0.34

The interval-based method has the highest mean Dice coefficient values (0.61±0.33) across evaluated language region and ranks highest for 10 out of the 25 language-related regions (highlighted in blue in Table 4). Mean Dice coefficient values for the interval-based method range from 0.26 (ON-P3) to 0.83 (SC-P3), with mean values larger or equal than 0.6 for most language-related regions except SC-P7, SC-P9, SWG-P3, ON-P3, AG-P7, AG-P8, SCL-P1, SCL-P2, SCL-P7, SCL-P8, indicating at least a moderate to high level of overlap between activated voxels identified by SPM and by the interval-based method. However, a high overlap (0.8-1.0) between activated voxels identified by SPM and by the interval-based method was only observed in 3 language-related regions (SC-P2, SC-P3, AG-P3). The frequency-based method had the lowest mean Dice coefficient (0.5±0.35) across the evaluated language regions and ranked the lowest for 13 out of 25 language-related regions. The frequency-based method achieved a mean Dice coefficient of larger than 0.6 in only 7 regions (SC-P1, SC-P2, SC-P3, SC-P5, AG-P1, AG-P2, AG-P3), with a high level of overlap not being observed in any of the language regions.

The mean Dice coefficient differed significantly between ML Categories (Kruskal-Wallis Test, H(6)=41.8, $p=2.00\times 10^{-7}$). The post hoc Dunn test revealed that the mean Dice coefficient of the interval-based method was significantly larger than that of the dictionary, frequency and DL-based methods but not significantly different from that of the remaining methods. The mean Dice coefficient for the frequency-based method was significantly lower than that of the GML, feature and interval-based methods but not significantly different from that of the remaining methods.

### 3.4 Euclidean distance between peaks

**Figure 6:**
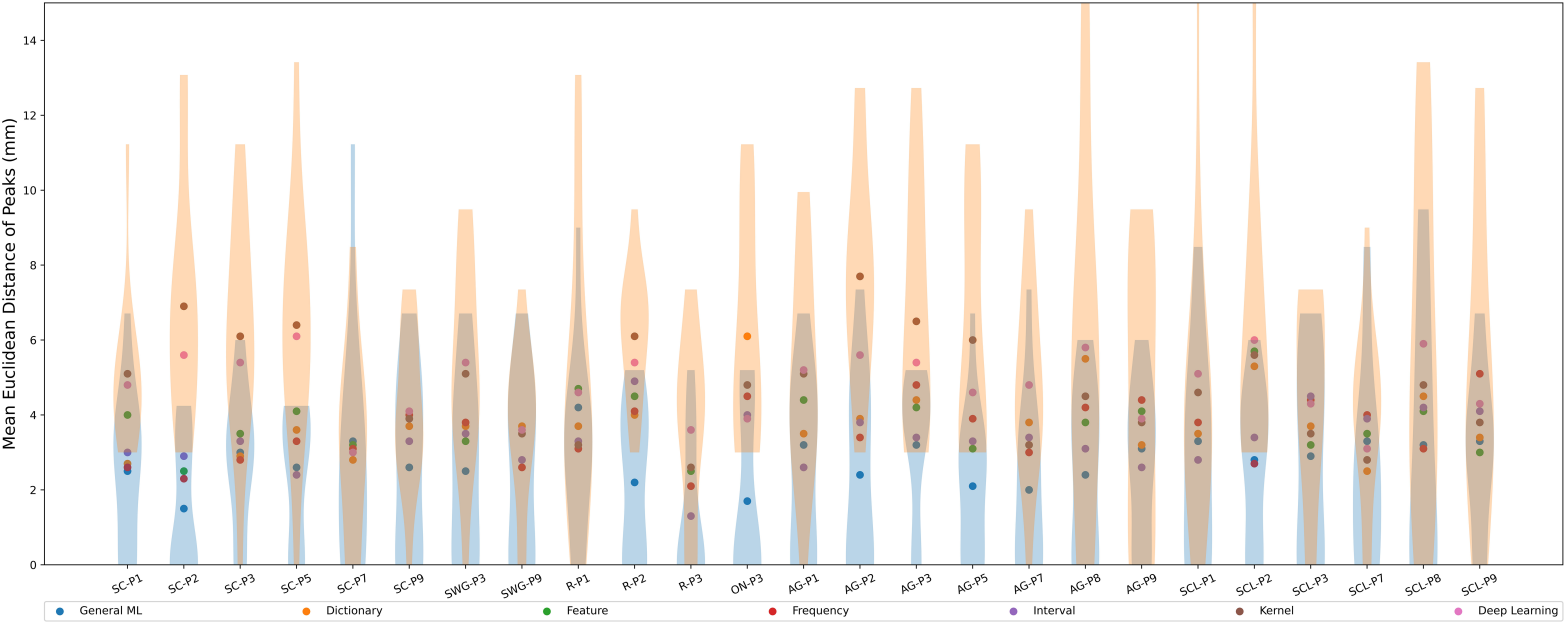
Mean Euclidean distance between activation peaks across test participants of different ML/DL categories and SPM, by language regions of interest (as a scatter plot with violin plots denoting the distribution of Euclidean distances for the best and worse performing ML/DL methods for each language paradigm. Blue - best, Orange - worst).

The scatter plot in Figure 6 shows the mean Euclidean distance (across test participants) between peaks in different language-related regions identified by SPM vs. peaks identified by the ML/DL methods in millimeters (mm). The violin plots show the distribution of Euclidean distances between peaks across test participants and ML/DL methods in different language-related regions. Table 5 shows the values associated with Figure 6.

**Table 5:** Table of mean Euclidean distance values across test participants by language regions of interest, associated with Figure 6.

	SC-P1	SC-P2	SC-P3	SC-P5	SC-P7	SC-P9	SWG-P3	SWG-P9	R-P1	R-P2	R-P3	ON-P3	AG-P1	AG-P2	AG-P3	AG-P5	AG-P7	AG-P8	AG-P9	SCL-P1	SCL-P2	SCL-P3	SCL-P7	SCL-P8	SCL-P9	Mean ± SD
General ML	2.5	1.5	3	2.6	3.3	2.6	2.5	2.6	4.2	2.2	2.5	1.7	3.2	2.4	3.2	2.1	2	2.4	3.1	3.3	2.8	2.9	3.3	3.2	3.3	2.7±2.4
Dictionary	2.7	5.6	2.9	3.6	2.8	3.7	3.7	3.7	3.7	4	2.6	6.1	3.5	3.9	4.4	4.6	3.8	5.5	3.2	3.5	5.3	3.7	2.5	4.5	3.4	3.9±2.9
Feature	4	2.5	3.5	4.1	3.2	3.9	3.3	2.6	4.7	4.5	2.5	4.8	4.4	3.4	4.2	3.1	3.2	3.8	4.1	2.8	5.7	3.2	3.5	4.1	3	3.7±3.0
Frequency	2.6	2.3	2.8	3.3	3.1	4	3.8	2.6	3.1	4.1	2.1	4.5	2.6	3.4	4.8	3.9	3	4.2	4.4	3.8	2.7	4.4	4	3.1	5.1	3.5±3.0
Interval	3	2.9	3.3	2.4	3	3.3	3.5	2.8	3.3	4.9	1.3	4	2.6	3.8	3.4	3.3	3.4	3.1	2.6	2.8	3.4	4.5	3.9	4.2	4.1	3.3±2.7
Kernel	5.1	6.9	6.1	6.4	3	3.9	5.1	3.5	3.2	6.1	2.6	4.8	5.1	7.7	6.5	6	3.2	4.5	3.8	4.6	5.6	3.5	2.8	4.8	3.8	4.7±3.1
Deep Learning	4.8	5.6	5.4	6.1	3	4.1	5.4	3.6	4.6	5.4	3.6	3.9	5.2	5.6	5.4	4.6	4.8	5.8	3.9	5.1	6	4.3	3.1	5.9	4.3	4.8±3.1

Euclidean distances ranged from 1.3 mm (R-P3, interval-based method) to 7.7 mm (AG-P2, kernel-based method) across different ML/DL categories. The GML method had the shortest average distance between SPM peaks and GML peaks for 13 out of 25 regions (highlighted in blue in Table 5), with distances ranging from 1.5 mm (SC-P2) to 4.2 mm (R-P1). The mean Euclidean distances between the peaks for the GML method was 2.7±2.4 mm. The DL and kernel-based methods ranked the lowest for 9 out of 25 and 8 out of 25 regions respectively. Euclidean distance for the DL-based method ranged from 3.0 mm (SC-P7) to 6.1 mm (SC-P5) and for the kernel-based method it ranged from 2.6 mm (R-P3) to 7.7 mm (AG-P2). The mean Euclidean distances for the DL and kernel-based method were 4.8±3.1 mm and 4.7±3.1 mm respectively.

The mean Euclidean distance differed significantly between ML categories (Kruskal-Wallis Test, H(6)=130.4, $p=1.04\times 10^{-}25$). On post hoc testing, the mean Euclidean distance for the GML method was significantly smaller than that of all other evaluated methods. The mean Euclidean distances for the kernel and DL-based methods did not significantly differ from each other but were significantly larger than that of all other evaluated methods.

Qualitative assessment of peaks was performed on the activation maps corresponding to the two most promising categories of methods (GML and interval-based). GML method identified peaks which were either in the same or adjacent gyrus to 80% of the peaks identified by SPM across the regions of interest. The interval-based method identified peaks in the same or adjacent gyrus to 60% of the peaks identified by SPM. (Note that in each participant, we only included language regions with at least 50% overlap and consequently the number of language regions of interest varied between test participants).

## 4 Discussion

We aimed to identify ML/DL methods for classification of language activation in fMRI time series. This was motivated by the challenges of analysing naturalistic fMRI data, where regressors are often difficult to define. fMRI data from seven language tasks were acquired. Experiments using task-based language activation data i.e., structured fMRI time series data, allowed us to understand how the ML/DL methods classify. We considered ML/DL methods for univariate time series classification from seven categories, namely general ML, DL, dictionary, feature, frequency, interval, and kernel. fMRI time series voxel data from 14 healthy participants were used for training (4,872 1D time series samples). Data from 12 participants including 6 healthy and 6 epilepsy patients were chosen for test. There were around 720,000 total 1D samples per language paradigm - see Appendix (Section A) for exact number of test samples. ML/DL models were trained on labelled data, using participant-specific SPM activation maps as the ground truth. The ML/DL methods were quantitatively evaluated using three different performance measures: whole-brain AUC, Dice coefficient and Euclidean distance between of activation peaks identified ML/DL and by SPM.

The GML and interval-based methods showed good correspondence with SPM activation (refer to Figure 3). Quantitatively, the GML method had the highest mean AUC values across the different ML/DL methods (0.97±0.03). Interestingly, whole-brain AUC values were high for all the evaluated ML/DL methods and the mean AUC values for the GML, feature, interval and kernel-based methods were not significantly different. The interval-based method achieved the highest mean Dice coefficient (i.e., 0.61±0.33). The GML method produced the smallest mean Euclidean distance for more than half the evaluated language regions, as well as the smallest mean distance (2.7±2.4 mm, superior by at least 0.5 mm compared to other ML/DL methods) when all of the evaluated language regions were considered. The GML and interval-based methods located peaks that were qualitatively similar in location to those identified by SPM for 80% (GML) and 60% of the (interval-based) evaluated peaks. The mean Euclidean distance for the GML method was significantly lower than for other evaluated methods. The DL and frequency-based methods rank lowest across evaluation metrics. While the frequency-based method showed excess areas of activation not corresponding to task activation, the DL-based method did predict reasonable activation. The methods associated with the other categories produced varied results, with no apparent trend in the metrics evaluated. Additionally, there was also no noticeable difference in results between healthy participants and epilepsy patients.

The results here suggest that the classification methods perform dissimilarly. Methods involving decision trees (GML and interval-based) outperform all other types of methods. Worst performing methods appeared to be those incorporating frequency information in one way or another into the features used for classification. The frequency-based method uses spectral features, and the DL-based method apply convolution operations (essentially a product operation in the frequency domain). Their inability to classify frequency features from the 1D time series information may be because the brain response during the task varies across time in a distinct, non-periodic, way (45). GML and the interval-based method rely on many decision trees chosen across the time series, essentially allowing selection of specific time points. The feature method also considers the time series features of the entire time series (38), which results in worse performance than GML and interval-based methods. Although not considered here, this suggests potential opportunities for reducing the length of the time series data without sacrificing classification accuracy.

A previous study using resting state fMRI data suggested that locations of abnormal brain activity could be predicted from 1D fMRI time series using a Convolutional Neural Network (CNN) (i.e., DL-based method) (46) and raised the possibility that DL-based methods can inform design of naturalistic fMRI stimuli. Our findings suggest that GML and interval-based methods may provide additional utility for 1D fMRI time series analysis and task design.

To our best knowledge, specific comparisons between methods categorised in the manner described here have not been performed to date, but comparisons of specific ML/DL methods in different research domains have been reported. Findings by Cabello et al. (40) on different types of time series data such as electrocardiographic recordings, stock market prices, seismic data, power demand over time, and other 1D time series data from the UCR database (47), suggest that the interval-based method sTSF is superior to TSF in terms of critical difference ranking (compared against 100+ datasets from the UCR database). This study informed our choice of sTSF in our study. Whilst not reported here, we did implement TSF and found sTSF to produce better results for time series fMRI data. In previous work, Bagnall et al. compared TSF with Rotation Forest (our GML method) and found TSF to be the best time series classification method (28), which were not concordant with our findings. In a subsequent study by Bagnall et al., the inception time approach, a DL algorithm and ROCKET, a kernel-based approach, sometimes did not perform well (48), perhaps because of over-fitting during training of these algorithms. This agrees with our finding that the DL-based method was inferior in classification performance to the others that we considered.

Our analyses indicate that, machine learning classification methods can be used to identify brain activation from fMRI time series data. The highlight the potential for ML/DL methods to identify activation in fMRI studies without pre-specified task regressors and in cognitive domains other than language.

### 4.1 Limitations and future work

Our study focused on comparing different ML/DL methods for the classification of task-based language fMRI time series but an analogous approach should be applicable to fMRI time series with a similar block design. We used default hyper-parameters for training the ML/DL methods and fMRI time series across different language tasks of similar block design and length were used (or the time series modified to ensure uniform length, such as padding for the AG task). Further work should examine how these methods generalize to other block designs or tasks (and fMRI time series without block designs such as naturalistic fMRI). This may involve identification of which time frame or combination of time frames within the fMRI time series contribute most to the classification resulting from the ML/DL method.

## 5 Conclusions

Our study involved seven routinely used fMRI language activation tasks. We evaluated the utility of different ML/DL methods from different time series classification algorithm categories in predicting which task-based language fMRI 1D time series data are activated by stimuli. The GML and interval-based method were able to best identify language areas and shows promise for use in fMRI data analysis. Our findings may lead to other work where the potential of machine learning approaches for 1D fMRI time series analysis are considered under different paradigms, such as visual and motor activation.

## Data Availability

The datasets presented in this article are not readily available as the data and code for this article is not publicly available due to patient privacy and ethical limitations. Requests to access the datasets should be directed to the corresponding author.

## Appendix

### A.1 Test set size for different participant and tasks

Refer to Table A1 for test set sizes of the various fMRI language tasks.

### A.2 Further justification of ML/DL choice

Authors in (28) suggest that on data that has never been evaluated before to start with Random Forest (RF), Rotation Forest (RotF) or Dynamic Time Warping (DTW) as a basic sanity check. Although DTW is known to be one of the more accurate algorithms, like other distance-based methods it is known to be slow and hard to scale (49) (For this reason the distance-based category was also excluded from further analysis). Hence, for the general ML category, RF and RotF were evaluated and RotF was retained due to its strong performance. For the dictionary-based category, two algorithms were short-listed, Temporal Dictionary Ensemble (TDE) and Word Extraction for Time Series Classification (WEASEL) (50). While TDE is built on the advantages of other dictionary-based algorithms such as Bag of Symbolic-Fourier Approximation (BOSS), Contractable Bag of Symbolic-Fourier Approximation (cBOSS) and Word Extraction for Time Series Classification (WEASLE)), TDE is known to be more accurate but also memory intensive (50). Authors in (50) also suggest that for faster prediction to use WEASLE. It was found that TDE indeed was memory intensive and took a long time to train, hence WEASLE was chosen instead. The Hierarchical Vote Collective of Transformation-Based Ensembles (HIVE-COTE) (51, 52) algorithm is an ensemble of 4 different algorithms (Shapelet Transform Classifier (STC), Time Series Forest (TSF), Random Interval Spectral Forest (RISE) and Contractable Bag of Symbolic-Fourier Approximation (cBOSS)) and was the early benchmark time series classifier with high accuracy. However, it had the disadvantage of long training and test times (28). It was since super-seeded by the Kernal-based method, RandOm Convolutional KErnel Transform (ROCKET) (53) algorithm (which was faster and more scalable) and subsequently HIVE-COTE version 2 (41). However, we found that the version 2 (v2) of HIVE-COTE (41) still has long training time and is also memory intensive, and was excluded from our analysis. The ROCKET algorithm fails to produce prediction probabilities, and ARSENAL which is an ensemble of ROCKET(s) was evaluated instead. The RISE algorithm (39) remains the only algorithm in the frequency-based category and was the only algorithm evaluated within the category. Several feature-based algorithms were also evaluated and the best performing algorithm, the Time Series Feature Extraction based on Scalable Hypothesis Tests (TSFresh) (38) method was retained. Several interval-based algorithms including Canonical Interval Forest (CIF) (54), Diverse Representation Canonical Interval Forest (DrCIF) (41) Time Series Forest (TSF) (55) and supervised Time Series Forest (sTSF) (40) were evaluated as task-based fMRI time series is block-designed and is known to have repetitive intervals. The best performing algorithm i.e., sTSF was retained. The shapelet based method, Shapelet Transform Classifier, was unable to generate prediction probabilities, and was excluded from further analysis. Deep learning algorithms have had great success in the image classification domain and efforts have been made to adapt these algorithms for time series classification. We found that Inception time performed well for time series classification compared to two other methods, Residual Neural Network (ResNet) and Fully Convolutional Network (FCN) and Inception Time was retained.

**Table A1:** Test set size for different participants and tasks.

	SC			SWG			R			ON			AG			PSL			SCL		
	0	1	Total	0	1	Total	0	1	Total	0	1	Total	0	1	Total	0	1	Total	0	1	Total
HC16	68,488	3,771	72,259	70,124	2,900	73,024	65,755	4,124	69,879	69,853	2,090	71,943	67,296	6,196	73,492	73,128	124	73,252	72,285	2,591	74,876
HC17	60,340	3,707	64,047	63,443	3,185	66,628	63,099	5,925	69,024	60,132	2,429	62,561	60,201	4,279	64,480	63,286	30	63,316	40,184	1,273	41,457
HC18	71,299	4,094	75,393	67,339	2,943	70,282	74,692	1,882	76,574	77,064	1,587	78,651	65,465	5,781	71,246	71,187	1,185	72,372	70,812	4,339	75,151
HC19	45,398	1,298	46,696	66,896	2,549	69,445	68,109	1,533	69,642	75,065	2,019	77,084	56,056	10,451	66,507	74,082	43	74,125	59,401	9,930	69,331
HC20	72,611	210	72,821	73,565	1,105	74,670	67,622	2,398	70,020	70,674	1,244	71,918	67,586	1,770	69,356	70,672	299	70,971	63,884	1,251	65,135
HC21	55,143	6,904	62,047	59,029	1,788	60,817	58,773	2,079	60,852	58,997	1,312	60,309	38,124	11,363	49,487	60,083	369	60,452	59,983	749	60,732
EP01	65,425	4,420	69,845	67,917	1,475	69,392	71,886	2,512	74,398	65,133	3,091	68,224	48,539	4,472	53,011	74,629	104	74,733	55,634	2,777	58,411
EP02	66,463	5,869	72,332	63,736	5,547	69,283	67,159	2,540	69,699	58,252	4,098	62,350	62,606	1,629	64,235	54,828	239	55,067	59,574	3,723	63,297
EP03	67,481	4,060	71,541	69,346	563	69,909	61,952	717	62,669	68,210	2,071	70,281	67,905	3,880	71,785	68,000	287	68,287	70,834	5,630	76,464
EP04	69,346	5,995	75,341	38,491	2,879	41,370	69,347	3,187	72,534	68,336	1,651	69,987	38,543	3,208	41,751	75,450	860	76,310	66,906	5,030	71,936
EP05	69,037	3,846	72,883	74,602	1,758	76,360	73,382	2,064	75,446	74,873	1,327	76,200	39,220	4,575	43,795	62,232	313	62,545	42,213	515	42,728
EP06	42,397	1,165	43,562	77,831	96	77,927	37,845	1,736	39,581	58,548	1,511	60,059	43,376	661	44,037	58,338	0	58,338	63,769	2,990	66,759
